# Hosoya Polynomials of Power Graphs of Certain Finite Groups

**DOI:** 10.3390/molecules27186081

**Published:** 2022-09-18

**Authors:** Bilal Ahmad Rather, Fawad Ali, Suliman Alsaeed, Muhammad Naeem

**Affiliations:** 1 Mathematical Sciences Department, College of Science, United Arab Emirate University, Al Ain 15551, United Arab Emirates; 2 School of Mathematics and Statistics, Xi’an Jiaotong University, Xi’an 710049, China; 3 Institute of Numerical Sciences, Kohat University of Science & Technology, Kohat 26000, Pakistan; 4 Applied Sciences College, Department of Mathematical Sciences, Umm Al-Qura University, P.O. Box 715, Makkah 21955, Saudi Arabia; 5 Deanship of Joint First Year, Umm Al-Qura University, Makkah 21955, Saudi Arabia

**Keywords:** finite groups, molecular structure, power graphs, Hosoya polynomial

## Abstract

Assume that G is a finite group. The power graph P(G) of G is a graph in which G is its node set, where two different elements are connected by an edge whenever one of them is a power of the other. A topological index is a number generated from a molecular structure that indicates important structural properties of the proposed molecule. Indeed, it is a numerical quantity connected with the chemical composition that is used to correlate chemical structures with various physical characteristics, chemical reactivity, and biological activity. This information is important for identifying well-known chemical descriptors based on distance dependence. In this paper, we study Hosoya properties, such as the Hosoya polynomial and the reciprocal status Hosoya polynomial of power graphs of various finite cyclic and non-cyclic groups of order pq and pqr, where p,q and r(p≥q≥r) are prime numbers.

## 1. Introduction

Quantitative structure–property relationships (QSPR) studies are provided by the physicochemical characteristics and topological indices, such as the atom–bond connectivity index, the geometric–arithmetic index, and the Randić index, which identify the bioactivity of chemical compounds. In fact, a topological index is created by converting a chemical structure (i.e., a graph) to a numerical value. It establishes relationships between various physicochemical properties of molecular structured chemical compounds, including the stability, the boiling point, and the strain energy. It is a numerical number that quantifies a molecular structure’s symmetries, defines its topology, and is constantly under a structure-preserving function [1]. Several topological indices may be used to explore specific properties of chemical substances with a microstructure. In 1947, Wiener developed the concept of the topological index, which he termed the *path number* while exploring the boiling point of paraffin [2]. As a consequence, it became known as the *Wiener index*, and this was the origin of the concept of topological indices. Numerous degree-based and distance-based topological indices have been presented and calculated in the past few years; for instance, see [3,4,5,6,7].

Various scientists utilized Pólya’s [8] concept of evaluating polynomials to determine the unsaturated hydrocarbon’s molecular orbital. The graph spectra have been widely studied in this context. Hosoya [9] employed this concept in 1988 to establish the polynomials of various chemical structures that became referred to as the *Hosoya polynomials* and garnered worldwide attention. This polynomial was called the Wiener polynomial by Sagan et al. [10] in 1996; however, several researchers referred to it as the Hosoya polynomial. The Hosoya polynomial provides details on graph invariants depending on the distance. In [11], Cash proposed a link amongst the Hosoya polynomial and the hyper Wiener index. Estrada et al. [12] examined several fascinating uses of the extended Wiener indices.

The graphs shown in this article are simple, without loops or multiple edges. Assume that G is a finite group. The power graph P(G) of G is a graph whose node set is G and two different elements are joined by an edge whenever one is the power of the other. Kelarev and Quinn gave the notion of directed power graphs concerning semigroups and groups [13]. Afterward, in [14], the authors demonstrated the P(S) of a semigroup S and defined the class of semigroups whose power graphs are complete. Additionally, they explained when the power graph of a group G is complete whenever the group G is cyclic of order prime power or one.

The power graph is a popular topic in various mathematics disciplines, including Lie algebra, ring theory, and group theory. The authors of [15] examined matching numbers and established upper and lower limits on the perfect matching of power graphs associated with specific groups. Furthermore, they demonstrated how to generate matching numbers for each finite nilpotent group. The authors of [16] focused on power indices graphs while categorizing all graphs into a few predefined groups. In [17], the authors investigated the greatest clique and discovered that power graphs have the maximum number of edges for every finite cyclic group. The node connectivity of $P(Z_{n})$, where *n* is the product of certain prime numbers, was studied in [18]. Additionally, several other scholars investigated other algebraic graph notions; for example, see [19,20,21,22,23,24,25,26,27].

Matching is the set of edges that do not intersect with any nodes. A node is said to be matched if it coincides with one of the matching edges. Alternatively, an unmatched node is present. The Hosoya index or *Z*-index indicates a graph’s greatest number of matchings. Hosoya [28] invented the *Z*-index in 1971 and later developed it to serve as a general mechanism for quantum chemistry [29]. It has now been proven to be extremely effective in various chemical issues, particularly the boiling point, entropy, and the heat of vaporization. Several researchers studied the extremal issues using the Hosoya index to use a variety of graph configurations. In [30,31,32,33,34,35], the excessive topological and Hosoya properties of various graphs, such as unicyclic graphs, Eulerian graphs, and trees were widely examined.

The Hosoya properties of general graphs are difficult to study, and providing exact formulae is very challenging. So, the authors restrict these properties to some classes of graphs and elaborate on various interesting properties, although gaps remain. Researchers [3,36,37,38,39] studied the topological indices such as the Hosoya polynomial of graphs defined on groups, such as fractal graphs, power graphs of finite groups, commuting, and non-commuting graphs of the group of symmetries. Calculating the (reciprocal) Hosoya polynomial of a power graph P(G) of an arbitrary group G is very complicated. Therefore, we have extended their work by finding the Hosoya as well as the reciprocal status Hosoya polynomials of power graphs of several finite groups of a different order.

The remaining article is organized as follows: Section 2 contains some relevant results and definitions useful for this paper. In Section 3, we explore the power graphs of finite cyclic as well as non-cyclic groups of order pq and pqr, whereas p,q and r(p≥q≥r) are different primes. Section 4 analyzes the reciprocal status Hosoya polynomial of power graphs of finite groups of order pq and pqr. Section 5 contains the conclusion of the paper.

## 2. Basic Notions and Notations

This section summarizes various basic graph–theoretic features and notable results that will be discussed later in the paper.

Assume that Γ is an undirected finite simple graph. The edge and node sets of Γ are indicated by E(Γ) and V(Γ), respectively. The *distance* from $u_{1}$ to $u_{2}$ in Γ symbolized by $\mathrm{dis}(u_{1},\;u_{2})$ is based on the length of the smallest path between them. The order of Γ is determined as the number of nodes, which is indicated by |Γ|. Two distinct nodes $v_{1}$ and $v_{2}$ are connected if they share an edge, and it is represented by $v_{1}∼v_{2}$; otherwise, $v_{1}≁v_{2}.$ The degree or valency of a node $u_{1}$ is $\deg(u_{1})$, which represents the set of nodes in Γ that are edge connected to $u_{1}$. A $u_{1}-u_{2}$ path having $\mathrm{dis}(u_{1},u_{2})$ length is known as a $u_{1}-u_{2}$ *geodesic*. The greatest distance between $u_{1}$ and other nodes in Γ is referred to as the *eccentricity* and is indicated by the symbol $\mathrm{ec}(u_{1})$. The *diameter* denoted by diam(Γ) of Γ is the greatest eccentricity amongst all the nodes of Γ. Additionally, the *radius* denoted by rad(Γ) is the least eccentricity of all the nodes in Γ.

Assume that Γ is a connected graph of degree n. Hosoya defines the polynomial of Γ as given below:

(1) $$H\left(\Gamma ,y\right)=\sum_{i\geq 0}\mathrm{dis}\left(\Gamma ,i\right)y^{i}.$$

The coefficient dis(Γ,i) denotes the total of (v,w) pairs of nodes such that dis(v,w)=i, where i≤diam(Γ). In [40], the authors presented the following reciprocal status Hosoya polynomial for Γ:

(2) $$H_{rs}\left(\Gamma ,y\right)=\sum_{vw\in E(\Gamma )}y^{rs(v)+rs(w)},$$

where $rs\left(w\right)=\sum_{v\in V(\Gamma ),v\neq w}\frac{1}{\mathrm{dis}(w,v)}$ is called the reciprocal status or the reciprocal transmission of *w*.

Assume that $\Gamma_{1}$ and $\Gamma_{2}$ are two graphs that are connected; then, $\Gamma_{1}\vee \Gamma_{2}$ is the join of $\Gamma_{1}$ and $\Gamma_{2}$ where the node and edge sets are $V\left(\Gamma_{1}\right)\cup V\left(\Gamma_{2}\right)$ and

$$E\left(\Gamma_{1}\right)\cup E\left(\Gamma_{2}\right)\cup \left\{y∼z:y\in V\left(\Gamma_{1}\right),\;z\in V\left(\Gamma_{2}\right)\right\},$$

respectively. An edge connecting any two nodes in a graph is known as a complete graph, and it is represented by $K_{n}$. Additional undefined terms and notations were obtained from [41,42].

We denote the cyclic group of order *n* by $Z_{n}$. In addition, the direct product of groups $G_{1},G_{2},\cdots ,G_{n}$ having binary operations ${⋆}_{1},{⋆}_{2},\cdots ,{⋆}_{n}$, respectively, is the collection of all ordered *n*-tuples $\left(x_{1},x_{2},\cdots ,x_{n}\right)$ component-wise operation defined by

$$\left(x_{1},x_{2},\cdots ,x_{n}\right)⋆\left(x_{1}^{\prime },x_{2}^{\prime },\cdots ,x_{n}^{\prime }\right)=\left(x_{1}{⋆}_{1}x_{1}^{\prime },x_{2}{⋆}_{2}x_{2}^{\prime },\cdots ,x_{n}{⋆}_{n}x_{n}^{\prime }\right),$$

where $x_{i}{⋆}_{i}x_{i}^{\prime }$ is the product in $G_{i}$ for each *i*. Similarly, for the definition of semidirect product of groups, see ([43], p. 177).

## 3. Hosoya Polynomials

The following result gives the structure of power graphs of finite groups of order pq.

**Lemma** **1** ([44]). *Assume that G is a finite group and |G|=pq, whereas p and q (p<q) are primes. Then, the subsequent properties hold.*

 **(i)** 
*$P\left(G\right)≅K_{pq-p-q+2}\vee \left(K_{p-1}\cup K_{q-1}\right)$ if and only if G is cyclic.*
 **(ii)** 
*$P\left(G\right)≅K_{1}\vee \left(\underset{q}{\underset{︸}{K_{p-1}\cup K_{p-1}\cup \cdots \cup K_{p-1}}}\cup K_{q-1}\right)$ if and only if G is non-cyclic.*

Next, the following result provides the Hosoya polynomial of power graphs of a finite group G of order pq.

**Theorem** **1.** *Suppose G is a finite group and |G|=pq,(p<q). Then, the subsequent holds.*

 **(i)** 
*If G is cyclic of order n=pq, then*
$$H\left(P(G),y\right)=\left(pq-p-q+1\right)y^{2}+\frac{1}{2}\left({\left(pq\right)}^{2}-3pq+2p+2q-2\right)y+pq.$$
 **(ii)** 
*If G is non-cyclic of order n=pq, then*
$$H\left(P(G),y\right)=\frac{q}{2}\left(p-1\right)\left(p+1\right)\left(q-1\right)y^{2}+\frac{q}{2}\left(p^{2}+q-p-1\right)y+pq.$$

**Proof.**  By the definition of Hosoya coefficients given in Equation (1), we need to determine dis(P(G),0),dis(P(G),1), and $\mathrm{dis}\left(P(G),2\right)$. Consider now a node set $V_{k}$ that contains any pair of P(G) nodes; then,

|Vk|=pq2+pq=pq(pq+1)2.

Suppose

$$C\left(P\left(G\right),\ell \right)=\left\{(j,k);j,k\in V(P(G))\;|\;\mathrm{dis}(j,k)=\ell \right\},$$

and dis(P(G),ℓ)=|C(P(G),ℓ)|. Then:

(3) $$V_{k}=C\left(P\left(G\right),0\right)\cup C\left(P\left(G\right),1\right)\cup C\left(P\left(G\right),2\right).$$

Since, for each j∈V(P(G)), dis(j,j)=0, so

$$C\left(P\left(G\right),0\right)=\left\{(j,j);j\in V(P(G))\right\},$$

and is equal to V(P(G)). Therefore, C(P(G),0)=n. Applying **(i)** of Lemma 1, we have $P\left(G\right)≅K_{pq-p-q+2}\vee \left(K_{p-1}\cup K_{q-1}\right)$ with $V\left(K_{pq-p-q+2}\right)=A_{1},$$V\left(K_{p-1}\right)=A_{2},$ and $V\left(K_{q-1}\right)=A_{3}.$ Therefore,

$$\begin{array}{cc} C(P(G),1)= & \left\{\left(j,k\right);j\in A_{1},k\in A_{2}\right\}\cup \left\{\left(j,k\right);j\in A_{1},k\in A_{3}\right\}\\ \; & \cup \left\{\left(j,k\right);k,j\in A_{1}\;\mathrm{and}\;k\neq j\right\}\cup \left\{\left(j,k\right);k,j\in A_{2}\;\mathrm{and}\;k\neq j\right\}\\ \; & \cup \left\{\left(j,k\right);k,j\in A_{3}\;\mathrm{and}\;k\neq j\right\}. \end{array}$$

Consequently,

C(P(G),1)=(2+pq−q−p)(p−1)+(2+pq−q−p)(q−1)+2+pq−q−p2+p−12+q−12=12(pq)2−3pq+2p+2q−2.

Using Equation (3), we obtain:

$$|V_{k}|=\mathrm{dis}\left(P(G),0\right)+\mathrm{dis}\left(P(G),1\right)+\mathrm{dis}\left(P(G),2\right).$$

Hence,

$$\begin{array}{cc} \mathrm{dis}\left(P(G),2\right)= & \;|V_{k}|-\mathrm{dis}\left(P(G),0\right)-\mathrm{dis}\left(P(G),1\right)\\ = & \;\frac{pq(pq+1)}{2}-pq-\frac{1}{2}\left({\left(pq\right)}^{2}-3pq+2p+2q-2\right)\\ = & \;pq-q-p+1. \end{array}$$

Now, by Equation (1) and using the above calculation, we obtain

$$H\left(P\left(G\right),y\right)=\left(pq-p-q+1\right)y^{2}+\frac{1}{2}\left({\left(pq\right)}^{2}-3pq+2p+2q-2\right)y+pq.$$

**(ii)** Using Lemma 1, the power graph of G is $P\left(G\right)≅K_{1}\vee \left(\underset{q}{\underset{︸}{K_{p-1}\cup K_{p-1}\cup \cdots \cup K_{p-1}}}\cup K_{q-1}\right).$ Let $V\left(K_{q-1}\right)=A_{1},$$V\left(K_{p-1}\right)=A_{2}$ and retaining other notation as given in (i), we obtain:

$$\begin{array}{cc} C(P(G),1)= & \left\{\left(j,k\right);k\in A_{1},j=e\right\}\cup \overset{q}{\underset{j=1}{⋃}}\left\{\left(j,k\right);k\in A_{2},j=e\right\}\\ \; & \cup \left\{\left(j,k\right);k\in A_{1},j=e\;\mathrm{and}\;k\neq j\right\}\cup \overset{q}{\underset{j=1}{⋃}}\left\{\left(j,k\right);k\in A_{2},j=e\;\mathrm{and}\;k\neq j\right\}. \end{array}$$

Thereby, it follows that

C(P(G),1)=q−1+q(p−1)+q−12+qp−12=q2p2+q−p−1.

Using Equation (3), we obtain

$$\begin{array}{cc} \mathrm{dis}\left(P(G),2\right)= & \;|V_{k}|-\mathrm{dis}\left(P(G),0\right)-\mathrm{dis}\left(P(G),1\right)\\ = & \;\frac{pq(pq+1)}{2}-pq-\frac{q}{2}\left(p^{2}+q-p-1\right)\\ = & \;\frac{q}{2}\left(q-1\right)\left(p+1\right)\left(p-1\right). \end{array}$$

Thus, the Hosoya polynomial of P(G) is given below:

$$H\left(P\left(G\right),y\right)=\frac{q}{2}\left(p-1\right)\left(p+1\right)\left(q-1\right)y^{2}+\frac{q}{2}\left(p^{2}+q-p-1\right)y+pq.$$

□

We denote G(p,q,r) as the class of all finite groups whose order is pqr, whereas p,q,r are primes. Hölder [45] (see, also [46]) investigated the structures of groups in G(p,q,r). For p=q=r, there are five groups of order $p^{3}$ that are given as:

$$\begin{array}{c} Z_{p}\times Z_{p^{2}},\;Z_{p}\times Z_{p}\times Z_{p},\;Z_{p}⋊Z_{p^{2}},\;Z_{p}⋊\left(Z_{p}\times Z_{p}\right),Z_{p^{3}}, \end{array}$$

where ⋊ is the semidirect product and × is the direct product of groups. For p>q>r, the groups of order pqr are given below:

- $Z_{pqr},$$F_{p,qr},\;\left(qr|p-1\right),$$Z_{p}\times F_{q,r},\;\left(r|q-1\right),$$Z_{r}\times F_{p,q},\;\left(q|p-1\right),$$Z_{q}\times F_{p,r},\;\left(r|p-1\right),$
- $G_{i+5}≅\langle \alpha ,\beta ,\gamma :\alpha^{p}=\beta^{q}=\gamma^{r}=1,\alpha \beta =\beta \gamma ,\gamma^{-1}\beta \gamma =\beta^{u},\gamma^{-1}\alpha \gamma =\alpha^{v^{i}}\rangle ,$ where q−1,r|p−1, o(u)=r in $Z_{q}^{*}$ and o(v)=r in $Z_{p}^{*}\;\left(1\leq i\leq r-1\right).$

Suppose for r=3, we have following groups of order 3pq, for $G_{i+5}.$

$G_{6}=\langle \alpha ,\beta ,\gamma :\alpha^{p}=\beta_{q}=\gamma^{3}=1,\alpha \beta =\beta \alpha ,\gamma^{-1}\beta \gamma =\beta^{u},\gamma^{-1}\alpha \gamma =\alpha^{v}\rangle ,$ where o(u)=3 in $Z_{q}^{*}$ and o(v)=3 in $Z_{p}^{*}.$

$G_{7}=\langle \alpha ,\beta ,\gamma :\alpha^{p}=\beta_{q}=\gamma^{3}=1,\alpha \beta =\beta \alpha ,\gamma^{-1}\beta \gamma =\beta^{u},\gamma^{-1}\alpha \gamma =\alpha^{v^{2}}\rangle ,$ where o(u)=3 in $Z_{q}^{*}$ and o(v)=3 in $Z_{p}^{*}.$

Based on these finite groups of order pqr, we find the Hosoya polynomials of their power graphs.

**Theorem** **2.** *The Hosoya polynomial of P(G) of the group $G=Z_{p}\times Z_{p^{2}}$ is given as:*

$$\begin{array}{c} H\left(P\left(G\right),y\right)=\frac{p}{2}\left(p^{3}+p^{2}+3p+1\right){\left(p-1\right)}^{2}y^{2}+\frac{p}{2}\left(p-1\right)\left(p^{3}-p^{2}+2p+1\right)y+p^{3}. \end{array}$$

**Proof.**  First, we find the Hosoya coefficients, dis(P(G),0),dis(P(G),1), and dis(P(G),2) of P(G). The node set $V_{k}$ for any pair of nodes of P(G) is given below:

|Vk|=p32+p3=p3(p3+1)2.

Clearly, C(P(G),0)=pqr. In addition, the structure of P(G) ( see [47]) is given as:

$$P\left(G\right)≅K_{1}\vee \left(\left(\underset{p-\mathrm{times}}{\underset{︸}{K_{p-1}\cup \cdots \cup K_{p-1}}}\right)⋃\left(K_{p-1}\vee \left(\underset{p-\mathrm{times}}{\underset{︸}{K_{p^{2}-p}\cup K_{p^{2}-p}\cup \cdots \cup K_{p^{2}-p}}}\right)\right)\right).$$

Suppose $V\left(K_{p-1}\right)=A_{1},\;V\left(K_{p^{2}-p}\right)=A_{2},$ and denote one of the remaining *p* copies of nodes of $V(K_{p-1})$ by $A_{3}$. Thus:

$$\begin{array}{cc} C(P(G),1)= & \{\left\{\left(j,k\right);k\in A_{1},j=e\right\}\cup \left\{\left(j,k\right);k\in A_{2},j=e\right\}\cup \left\{\left(j,k\right);k\in A_{3},j=e\right\}\\ \; & \cup \left\{\left(j,k\right);k,j\in A_{1}\;\mathrm{and}\;k\neq j\right\}\cup \left(\overset{p}{\underset{j=1}{⋃}}\left\{\left(j,k\right);k,j\in A_{2}\;\mathrm{and}\;k\neq j\right\}\right)\\ \; & \cup \left(\overset{p}{\underset{j=1}{⋃}}\left\{\left(j,k\right);k,j\in A_{3}\;\mathrm{and}\;k\neq j\right\}\right)\}. \end{array}$$

Consequently,

C(P(G),1)=p−1+p(p2−p)+p(p−1)+p−12+pp2−p2+pp−12=p2(p−1)(p3−p2+2p+1).

Therefore, from

$$|V_{k}|=\mathrm{dis}\left(P(G),0\right)+\mathrm{dis}\left(P(G),1\right)+\mathrm{dis}\left(P(G),2\right),$$

we obtain

$$\begin{array}{cc} \mathrm{dis}\left(P(G),2\right)= & \;|V_{k}|-\mathrm{dis}\left(P(G),0\right)-\mathrm{dis}\left(P(G),1\right)\\ = & \;\frac{p^{3}\left(1+p^{3}\right)}{2}-p^{3}-\frac{p}{2}\left(p-1\right)\left(p^{3}-p^{2}+2p+1\right)\\ = & \;\frac{p}{2}\left(p^{3}+p^{2}+3p+1\right){\left(p-1\right)}^{2}. \end{array}$$

By using the above values in Equation (1), we obtain

$$H\left(P\left(G\right),y\right)=\frac{p}{2}\left(p^{3}+p^{2}+3p+1\right){\left(p-1\right)}^{2}y^{2}+\frac{p}{2}\left(p^{3}-p^{2}+2p+1\right)y+p^{3}\left(p-1\right).$$

□

Proceeding in the same manner as in Theorem 2 and noticing that (see [47]) $P\left(Z_{p}\times Z_{p}\times Z_{p}\right)=K_{1}\vee \left(\overset{p^{2}+p+1}{\underset{i=1}{⋃}}K_{p-1}\right)$, we have the following result for the power graph of $Z_{p}\times Z_{p}\times Z_{p}.$

**Theorem** **3.** *The Hosoya polynomial of P(G) of $G=Z_{p}\times Z_{p}\times Z_{p}$ is given as:*

$$\begin{array}{c} H\left(P\left(G\right),y\right)=\frac{p}{2}\left(p+1\right){\left(p-1\right)}^{2}\left(1+p+p^{2}\right)y^{2}+\frac{p}{2}\left(p-1\right)\left(p^{2}+p+1\right)y+p^{3}. \end{array}$$

Let $G≅\langle x,y:x^{p^{2}}=y^{p}=1,y^{-1}xy=x^{p+1}\rangle .$ Then, for p≠2,

$$P\left(G\right)≅P\left(Z_{p}⋊Z_{p^{2}}\right)≅P\left(Z_{p}\times Z_{p^{2}}\right)$$

(see [47] Theorem 3.5), and its Hosoya polynomial is given in Theorem 2. For p=2, $P\left(G\right)≅P\left(Z_{2}⋊Z_{2^{2}}\right)=K_{1}\vee \left(K_{3}\cup {\bar{K}}_{4}\right),$ then its Hosoya polynomial is

(4) $$18y^{2}+10y+8.$$

Suppose $G≅\langle a,b,c:a^{p}=b^{p}=c^{p}=1,ac=cab,bc=cb,ab=ba\rangle .$ Then, for p=2,

$$P\left(G\right)≅P\left(Z_{p}\times Z_{p}\times Z_{p}\right)≅P\left(Z_{p}⋊\left(Z_{p}\times Z_{p}\right)\right),$$

and Theorem 3 specifies its Hosoya polynomial. For p=2,

$$P\left(G\right)≅P\left(Z_{2}⋊\left(Z_{2}\times Z_{2}\right)\right)≅P\left(Z_{2}⋊Z_{4}\right),$$

and its Hosoya polynomial is given as in Equation (4).

**Theorem** **4.** *The Hosoya polynomial of $P(Z_{pqr})$ of $Z_{pqr}$, where p,q,r are distinct primes is given as:*

$$\begin{array}{cc} H(P\left(Z_{pqr}\right),y)= & (3-3p+p^{2}-3q+3pq-p^{2}q+q^{2}-pq^{2}-3r+3pr-p^{2}r+3qr-3pqr\\ \; & +p^{2}qr-q^{2}r+pq^{2}r+r^{2}-pr^{2}-qr^{2}+pqr^{2})y^{2}+\frac{1}{2}(p^{2}q^{2}r^{2}-2p^{2}qr+2p^{2}q\\ \; & +2p^{2}r-2p^{2}-2pq^{2}r+2pq^{2}-2pqr^{2}+5pqr-6pq+2pr^{2}-6pr+6p\\ \; & +2q^{2}r-2q^{2}+2qr^{2}-6qr+6q-2r^{2}+6r-6)y+pqr. \end{array}$$

**Proof.**  Since the node set $V_{k}$ for any pair of nodes of $P(Z_{pqr})$ is:

|Vk|=pqr2+pqr=pqr(pqr+1)2.

Clearly, $C(P\left(Z_{pqr}\right),0)=pqr.$ The structure of $P(Z_{pqr})$ ([47]) is:

$$P\left(Z_{pqr}\right)≅K_{(p-1)(q-1)(r-1)+1}\vee C_{6}\left[K_{q-1},K_{qr-p-r+1},K_{r-1},K_{pr-p-r+1},K_{p-1},K_{pq-p-q+1}\right],$$

where $C_{6}$ is the cycle of order 6. Using node partitions of $P(Z_{pqr})$ as:

$$V\left(K_{(p-1)(q-1)(r-1)+1}\right)=A_{1},\;V\left(K_{q-1}\right)=A_{2},\;V\left(K_{qr-q-r+1}\right)=A_{3},\;V\left(K_{r-1}\right)=A_{4},$$

$$V\left(K_{pr-p-r+1}\right)=A_{5},\;V\left(K_{p-1}\right)=A_{6}\;\mathrm{and}\;V\left(K_{pq-p-q+1}\right)=A_{7}.$$

Thus:

$$\begin{array}{cc} C(P\left(Z_{pqr}\right),1)= & \left\{\left(j,k\right);k,j\in A_{1}\;\mathrm{and}\;j\neq k\right\}\cup \left\{\left(j,k\right);j\in A_{1},k\in A_{2}\right\}\cup \left\{\left(j,k\right);j\in A_{1},k\in A_{3}\right\}\\ \; & \cup \left\{\left(j,k\right);j\in A_{1},k\in A_{4}\right\}\cup \left\{\left(j,k\right);j\in A_{1},k\in A_{5}\right\}\cup \left\{\left(j,k\right);j\in A_{1},k\in A_{6}\right\}\\ \; & \cup \left\{\left(j,k\right);j\in A_{1},k\in A_{7}\right\}\cup \left\{\left(j,k\right);j,k\in A_{2}\;\mathrm{and}\;j\neq k\right\}\\ \; & \cup \left\{\left(j,k\right);j,k\in A_{3}\;\mathrm{and}\;j\neq k\right\}\cup \left\{\left(j,k\right);j,k\in A_{4}\;\mathrm{and}\;j\neq k\right\}\\ \; & \cup \left\{\left(j,k\right);j,k\in A_{5}\;\mathrm{and}\;j\neq k\right\}\cup \left\{\left(j,k\right);j,k\in A_{6}\;\mathrm{and}\;j\neq k\right\}\\ \; & \cup \left\{\left(j,k\right);j,k\in A_{7}\;\mathrm{and}\;j\neq k\right\}\cup \left\{\left(j,k\right);j\in A_{2},k\in A_{3}\right\}\cup \left\{\left(j,k\right);j\in A_{3},k\in A_{4}\right\}\\ \; & \cup \left\{\left(j,k\right);j\in A_{4},k\in A_{5}\right\}\cup \left\{\left(j,k\right);j\in A_{5},k\in A_{6}\right\}\cup \left\{\left(j,k\right);j\in A_{6},k\in A_{7}\right\}\\ \; & \cup \left\{\left(j,k\right);j\in A_{7},k\in A_{2}\right\}. \end{array}$$

Therefore,

C(P(Zpqr),1)=1+(p−1)(q−1)(r−1)2+1+(p−1)(q−1)(r−1)(q−1+qr−r−q+1+r−1+pr−p−r+1+p−1+pq−p−q+1)+q−12+r−12+qr−q−r+12+pr−p−r+12+p−12+pq−p−q+12+(q+r−2)(1+rq−r−q)+(1+rp−r−p+1)(p+r−2)+(1+qp−p−q)(p+q−2).=12(p2q2r2−2p2qr+2p2q+2p2r−2p2−2pq2r+2pq2−2pqr2+5pqr−6pq+2pr2−6pr+6p+2q2r−2q2+2qr2−6qr+6q−2r2+6r−6).

In addition,

$$\begin{array}{cc} \mathrm{dis}\left(P(Z_{pqr}),2\right)= & \;|V_{k}|-\mathrm{dis}\left(P(Z_{pqr}),0\right)-\mathrm{dis}\left(P(Z_{pqr}),1\right)\\ = & \;\frac{pqr(pqr+1)}{2}-pqr-\frac{1}{2}(p^{2}q^{2}r^{2}-2p^{2}qr+2p^{2}q+2p^{2}r-2p^{2}-2pq^{2}r\\ \; & +2pq^{2}-2pqr^{2}+5pqr-6pq+2pr^{2}-6pr+6p+2q^{2}r-2q^{2}+2qr^{2}-6qr\\ \; & +6q-2r^{2}+6r-6).\\ = & \;3-3p+p^{2}-3q+3pq-p^{2}q+q^{2}-pq^{2}-3r+3pr-p^{2}r+3qr-3pqr\\ \; & +p^{2}qr-q^{2}r+pq^{2}r+r^{2}-pr^{2}-qr^{2}+pqr^{2}. \end{array}$$

By inserting the aforementioned values into Equation (1), we obtain the essential Hosoya polynomial. □

**Figure 1:**
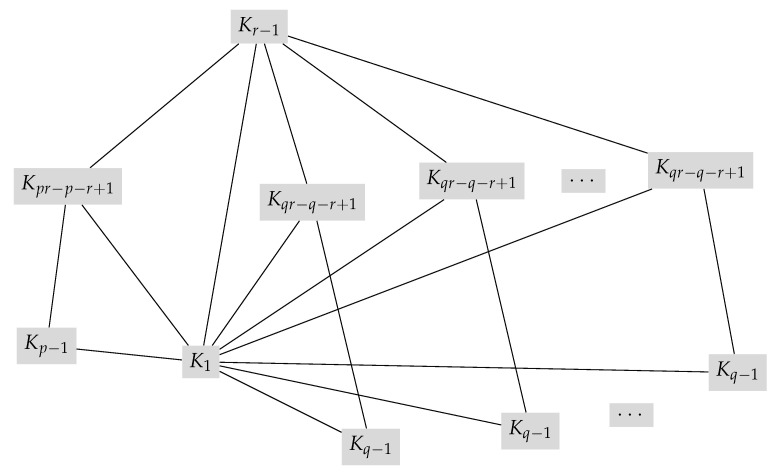
$P\left(Z_{r}\times F_{p,q}\right),\;\left(p≅1\left(\mathrm{mod}\;q\right)\right)$.

Next, we find the Hosoya polynomial of $P\left(Z_{r}\times F_{p,q}\right),\;\left(p≅1\left(\mathrm{mod}\;q\right)\right)$. Similarly, the Hosoya polynomials of $P\left(Z_{p}\times F_{q,r}\right),\;\left(q≅1\left(\mathrm{mod}\;r\right)\right)$ and $P\left(Z_{q}\times F_{p,r}\right),\;\left(p≅1\left(\mathrm{mod}\;r\right)\right)$ can be obtained. The power graph of $P\left(Z_{r}\times F_{p,q}\right),\;\left(p≅1\left(\mathrm{mod}\;q\right)\right)$ (see [47], Theorem 3.10) as a joined union is shown in Figure 1, where $K_{qr-q-r+1}$ and $K_{q-1}$ both occur *p*-times.

**Theorem** **5.** *The Hosoya polynomial of P(G) of the group $G=Z_{r}\times F_{p,q}$ is given as:*

$$\begin{array}{cc} H(P(G),y)= & \;\frac{p}{2}\left(pq^{2}r^{2}-pr^{2}-q^{2}r^{2}+2qr-2q+r^{2}\right)y^{2}+\frac{p}{2}\left(pr^{2}+q^{2}r^{2}-3qr+2q-r^{2}\right)y\\ \; & \;+pqr. \end{array}$$

**Proof.**  From Figure 1, consider the node partitions of P(G) as:

$V\left(K_{1}\right)=\left\{e\right\},\;V\left(K_{r-1}\right)=A_{1},\;V\left(K_{pr-p-r+1}\right)=A_{2},$$V\left(K_{r-1}\right)=A_{3},$$V\left(K_{qr-q-r+1}\right)=A_{4},\;\mathrm{and}\;V\left(K_{q-1}\right)=A_{5}.$ Thus:

$$\begin{array}{cc} C(P(G),1)= & \left\{\left(j,k\right);j=e,k\in A_{1}\right\}\cup \left\{\left(j,k\right);j=e,k\in A_{2}\right\}\cup \left\{\left(j,k\right);j=e,k\in A_{3}\right\}\\ \; & \cup \left(\overset{p}{\underset{j=1}{⋃}}\left\{\left(j,k\right);j=e,k\in A_{4}\right\}\right)\cup \left(\overset{p}{\underset{j=1}{⋃}}\left\{\left(j,k\right);j=e,k\in A_{5}\right\}\right)\\ \; & \cup \left\{\left(j,k\right);k,j\in A_{1},\;\mathrm{and}\;k\neq j\right\}\cup \left\{\left(j,k\right);k,j\in A_{2},\;\mathrm{and}\;k\neq j\right\}\\ \; & \cup \left\{\left(j,k\right);k,j\in A_{3}\;\mathrm{and}\;k\neq j\right\}\cup \left\{\left(j,k\right);k,j\in A_{3}\;\mathrm{and}\;k\neq j\right\}\\ \; & \cup \left(\overset{p}{\underset{j=1}{⋃}}\left\{\left(j,k\right);k,j\in A_{4}\;\mathrm{and}\;k\neq j\right\}\right)\cup \left(\overset{p}{\underset{j=1}{⋃}}\left\{\left(j,k\right);k,j\in A_{5}\;\mathrm{and}\;k\neq j\right\}\right)\\ \; & \cup \left(\overset{p}{\underset{j=1}{⋃}}\left\{\left(j,k\right);j\in A_{3},k\in A_{4}\right\}\right)\cup \left(\overset{p}{\underset{j=1}{⋃}}\left\{\left(j,k\right);k,j\in A_{4},k\in A_{5}\right\}\right). \end{array}$$

From the above computation, we obtain:

C(P(G),1)=p−1+rp−p−r+1+r−1+p(1+rq−r−q)+p(q−1)+p−12+pr−r−p+12+r−12+(rp−p−r+1)(p+r−2)+pq−12+pqr−q−r+12+qp−q−p+12+(rq−q−r+1)(rp+qp−2p)=12ppr2+q2r2−3qr+2q−r2.

In addition,

$$\begin{array}{cc} \mathrm{dis}\left(P(G),2\right)= & \;|V_{k}|-\mathrm{dis}\left(P(G),0\right)-\mathrm{dis}\left(P(G),1\right)\\ = & \;\frac{pqr(pqr+1)}{2}-pqr-\frac{1}{2}p\left(pr^{2}+q^{2}r^{2}-3qr+2q-r^{2}\right)\\ = & \;\frac{1}{2}p\left(pq^{2}r^{2}-pr^{2}-q^{2}r^{2}+2qr-2q+r^{2}\right). \end{array}$$

Therefore, the Hosoya polynomial of P(G) is given as follows:

$$\begin{array}{cc} H(P(G),y)= & \frac{p}{2}\left(pq^{2}r^{2}-pr^{2}-q^{2}r^{2}+2qr-2q+r^{2}\right)y^{2}+\frac{p}{2}\left(pr^{2}+q^{2}r^{2}-3qr+2q-r^{2}\right)y+pqr. \end{array}$$

□

Now, we examine the Hosoya polynomial of the power graph of $F_{p,qr}\;\left(p≅1(\mathrm{mod}\;qr)\right).$ The structure of $P(F_{p,qr})$ is given in [47] (see Theorem 3.12). The following results can be proved in the same manner as Theorems 2 and 5.

**Theorem** **6.** *Suppose $G≅F_{p,qr}\;\left(p≅1(mod\;qr)\right)$ is the group of order pqr. Then, the following characteristics hold true.*

 **(i)** 
*For r=3 or q=3, the Hosoya polynomial of P(G) is given below:*
$$\begin{array}{cc} H(P(G),y)= & \;\frac{1}{2}\left(p-1\right)\left(pq^{2}r^{2}-pr^{2}+2r-2\right)y^{2}+\frac{1}{2}(p^{2}r^{2}+pq^{2}r^{2}-pqr-pr^{2}-2pr\\ \; & +2p+2r-2)y+pqr. \end{array}$$
 **(ii)** 
*When r,q≠3, then the Hosoya polynomial of P(G) is given below:*
$$\begin{array}{cc} H(P(G),y)= & \;\frac{1}{2}p\left(qr-1\right)\left(qr+1\right)\left(p-1\right)y^{2}+\frac{1}{2}p\left(p+q^{2}r^{2}-qr-1\right)y+pqr. \end{array}$$

The following result calculates the Hosoya polynomial of the power graph of $G_{i+5}$, and its proof is similar to the proof of the above results.

**Theorem** **7.** *The following is the Hosoya polynomial of $P(G_{i+5})$:*

$$\begin{array}{cc} H(P\left(G_{i+5}\right),y)= & \;\frac{1}{2}\left(p^{2}q^{2}r^{2}-p^{2}q^{2}-pqr^{2}+3pq-2p-2q+2\right)y^{2}+\frac{1}{2}(p^{2}q^{2}+pqr^{2}-pqr\\ \; & -3pq+2p+2q-2)y+pqr. \end{array}$$

## 4. Reciprocal Status Hosoya Polynomials

The reciprocal status Hosoya polynomials of power graphs of finite groups pq and pqr will be determined in this section.

The first result establishes the reciprocal status Hosoya polynomials on the power graphs of cyclic and non-cyclic groups whose orders are the product of two different primes.

**Theorem** **8.** *Suppose G is a finite group of order qp,(q>p). Then, the following characteristics hold true.*

 **(i)** 
*If G is cyclic and |G|=pq, then*
$$\begin{array}{cc} H_{rs}\left(P\left(G\right),y\right)= & \;\left(2+qp-q-p\right)\left(q-1\right)y^{\frac{4pq-q-3}{2}}+\left(q-1\right)\left(2+qp-q-p\right)y^{\frac{4pq-p-3}{2}}\\ \; & \;+\frac{\left(1+qp-q-p)(2+qp-q-p\right)}{2}y^{2(pq-1)}+\frac{\left(p-2)(p-1\right)}{2}y^{2pq-q-1}\\ \; & \;+\frac{\left(p-1)(p-2\right)}{2}y^{2pq-p-1}. \end{array}$$
 **(ii)** 
*If G is non-cyclic and |G|=pq, then*
$$\begin{array}{cc} H_{rs}\left(P\left(G\right),y\right)= & \;\left(q-1\right)y^{\frac{3pq+q-4}{2}}+q\left(p-1\right)y^{\frac{4pq-3q-1}{2}}+\frac{\left(q-2)(q-1\right)}{2}y^{pq+q-2}\\ \; & +\frac{q(p-2)(p-1)}{2}y^{2pq-3q+1}. \end{array}$$

**Proof.**  Using Lemma 1, the power graph of the cyclic group G is given below:

$$P\left(G\right)≅K_{pq-p-q+2}\vee \left(K_{p-1}\cup K_{q-1}\right),$$

with node partition sets $V\left(K_{pq-p-q+2}\right)=A_{1},V\left(K_{p-1}\right)=A_{2}$ and $V\left(K_{q-1}\right)=A_{3}$. So, when $v\in A_{1}$, then ec(v)=1; also, we use the reciprocal status idea, resulting in the following:

rs(v)=pq−1.

When $v\in A_{2}$, then ec(v)=2. Additionally, we use the reciprocal status idea, resulting in the following:

$$rs\left(v\right)=p-2+qp-q-p+2+\frac{q-1}{2}=\frac{2pq-q-1}{2}.$$

When $v\in A_{3}$, implying ec(v)=2, further, we use the idea of reciprocal status, resulting in the following:

$$rs\left(v\right)=q-2+pq-p-q+2+\frac{p-1}{2}=\frac{2pq-p-1}{2}.$$

Clearly, from the structure of P(G), there are five distinct kinds of edges in P(G), namely: u∼v,u∼w,u∼u,v∼v and w∼w, where we take u=pq−1,$v=\frac{2pq-q-1}{2}$ and $w=\frac{2pq-p-1}{2}.$ Thus, using the reciprocal status Hosoya polynomial, we obtain:

(5) $$H_{rs}\left(P\left(G\right),y\right)=\sum_{E_{u∼v}}y^{u+v}+\sum_{E_{u∼w}}y^{u+w}+\sum_{E_{u∼u}}y^{2u}+\sum_{E_{v∼v}}y^{2v}+\sum_{E_{w∼w}}y^{2w}.$$

In addition, the edge set of type u∼v is, $E_{u∼v}=\left\{ab\in E\left(P\left(G\right)\right):rs\left(a\right)=u,rs\left(b\right)=v\right\}$ and the order of $E_{u∼v}$ is $|E_{u∼v}|=\left(pq-p-q+2\right)\left(q-1\right).$ Similarly, $|E_{u∼u}|=\frac{\left(2+qp-q-p)(1+qp-q-p\right)}{2},$$|E_{u∼w}|=\left(2+qp-q-p\right)\left(q-1\right),$$|E_{v∼v}|=\frac{\left(p-2)(p-1\right)}{2},$ and $|E_{w∼w}|=\frac{\left(p-2)(p-1\right)}{2}.$ Substituting all these values in Equation (5), we obtain:

$$\begin{array}{cc} H_{rs}\left(P\left(G\right),y\right)= & \;\left(q-1\right)\left(2+pq-q-p\right)y^{\frac{4pq-q-3}{2}}+\left(pq-p-q+2\right)\left(q-1\right)y^{\frac{4pq-p-3}{2}}\\ \; & \;+\frac{\left(1+qp-q-p)(2+qp-q-p\right)}{2}y^{2(pq-1)}+\frac{\left(p-2)(p-1\right)}{2}y^{2pq-q-1}\\ \; & \;+\frac{\left(p-1)(p-2\right)}{2}y^{2pq-p-1}. \end{array}$$

(ii) By Lemma 1, $P\left(G\right)≅K_{1}\vee \left(\underset{q}{\underset{︸}{K_{p-1}\cup \cdots \cup K_{p-1}}}\cup K_{q-1}\right),$ with $V\left(K_{1}\right)=\left\{e\right\},$$V\left(K_{p-1}\right)=A_{2}$ and $V\left(K_{q-1}\right)=A_{3}$.

So, when $v\in V(K_{1})$, then ec(v)=1, and proceeding as in (i), we have:

rs(v)=pq−1.

When $v\in A_{2}$, then ec(v)=2, and using the idea of reciprocal status, resulting in the following:

$$rs\left(v\right)=q-2+1+\frac{q(p-1)}{2}=\frac{pq+q-2}{2}.$$

When $v\in A_{3}$, implying ec(v)=2. Additionally, we incorporate the notion of reciprocal status, which results in the following:

$$rs\left(v\right)=q\left(p-2\right)+1+\frac{q-1}{2}=\frac{2pq-3q+1}{2}.$$

Furthermore, from the structure of P(G), we see that there are four distinct kinds of edges, namely: u∼w,v∼v,u∼v, and w∼w, where we let u=pq−1,$v=\frac{pq+q-2}{2}$ and $w=\frac{2pq-3q+1}{2}.$ Therefore, by Equation (2), we have

(6) $$H_{rs}\left(P\left(G\right),y\right)=\sum_{E_{u∼v}}y^{u+v}+\sum_{E_{u∼w}}y^{u+w}+\sum_{E_{v∼v}}y^{2v}+\sum_{E_{w∼w}}y^{2w}.$$

In addition, the cardinality of the corresponding edge sets is $|E_{u∼v}\left|=q-1,\;\right|E_{u∼w}|=q\left(p-1\right),$$|E_{v∼v}|=\frac{\left(q-1)(q-2\right)}{2},$ and $|E_{w∼w}|=\frac{q(p-1)(p-2)}{2}.$ Putting these values in Equation (5), we obtain:

$$\begin{array}{cc} H_{rs}\left(P\left(G\right),y\right)= & \;\left(q-1\right)y^{\frac{3pq+q-4}{2}}+q\left(p-1\right)y^{\frac{4pq-3q-1}{2}}+\frac{\left(q-2)(q-1\right)}{2}y^{pq+q-2}\\ \; & +\frac{q(p-2)(p-1)}{2}y^{2pq-3q+1}. \end{array}$$

□

Next, we calculate the reciprocal status Hosoya polynomials of all those groups whose order is $p^{3}$.

**Theorem** **9.** *Assume that P(G) is the power graph of $G=Z_{p}\times Z_{p^{2}}$ order $p^{3}.$ Then*

Hrs(P(G),y)=(p−1)y4p3−p2+p−42+p(p2−p)y4p3+p2−42+p(p−1)y2p3+p2+p−42+p−12yp3+p2−2+p(p−1)(p2−p)y3p3+p−42+pp2−p2yp3+p2−2+pp−12yp2+p−2.

**Proof.**  Using the node partitions of P(G) as given in Theorem 2 and $V\left(K_{1}\right)=\left\{e\right\}$, we have: For v=e, then ec(v)=1. Additionally, we use the idea of reciprocal status, resulting in the following:

$$rs\left(v\right)=p\left(p-1\right)+p-1+p\left(p^{2}-p\right)=p^{3}-1.$$

For $v\in A_{1}$, ec(v)=2. Additionally, we incorporate the notion of reciprocal status, which results in the following:

$$rs\left(v\right)=\frac{p(p-1)}{2}+\left(p-1\right)+p\left(p^{2}-p\right)=\frac{2p^{3}-p^{2}+p-2}{2}.$$

When $v\in A_{2}$, then ec(v)=2. Furthermore, we incorporate the notion of reciprocal status, which results in the following:

$$rs\left(v\right)=p^{2}-p-1+1+p-1+\frac{1}{2}\left(p\left(p-1\right)+\left(p^{2}-p\right)\left(p-1\right)\right)=\frac{p^{3}+p^{2}-2}{2}.$$

When $v\in A_{3}$, then ec(v)=2. Furthermore, using the idea of reciprocal status results in the following:

$$rs\left(v\right)=\left(p-1\right)+\frac{1}{2}\left({\left(p-1\right)}^{2}+p\left(p^{2}-p\right)+p-1\right)=\frac{p^{3}+p-2}{2}.$$

From the structure of P(G), we see that there are six kinds of edges in P(G), namely: u∼v,u∼w,u∼x,v∼v,v∼w,w∼w and x∼x, where we take $u=p^{3}-1,$$v=\frac{2p^{3}-p^{2}+p-2}{2},\;w=\frac{p^{3}+p^{2}-2}{2},$ and $x=\frac{p^{2}+p-2}{2}.$ Therefore,

(7) $$H_{rs}\left(P\left(G\right),y\right)=\sum_{E_{u∼v}}y^{u+v}+\sum_{E_{u∼w}}y^{u+w}+\sum_{E_{u∼u}}y^{2u}+\sum_{E_{v∼v}}y^{2v}+\sum_{E_{w∼w}}y^{2w}.$$

In addition, the edge set of type u∼v is $E_{u∼v}=\left\{ab\in E\left(P\left(G\right)\right):rs\left(a\right)=u,rs\left(b\right)=v\right\}$ and the order of $E_{u∼v}$ is $|E_{u∼v}|=p-1.$ Similarly, $|E_{u∼w}|=p\left(p^{2}-p\right),$$|E_{u∼x}|=p\left(p-1\right),$|Ev∼v|=p−12,$|E_{v∼w}|=p\left(p-1\right)\left(p^{2}-p\right),$|Ew∼w|=pp2−p2, and |Ex∼x|=pp−12. Substituting all these values in Equation (7), we have:

Hrs(P(G),y)=(p−1)y4p3−p2+p−42+p(p2−p)y4p3+p2−42+p(p−1)y2p3+p2+p−42+p−12yp3+p2−2+p(p−1)(p2−p)y3p3+p−42+pp2−p2yp3+p2−2+pp−12yp2+p−2.

□

We obtain the following result by performing the processes described in Theorem 9.

**Theorem** **10.** *Consider P(G) is the power graph of $G=Z_{p}\times Z_{p}\times Z_{p}$ of order $p^{3}.$ Then*

Hrs(P(G),y)=(p3−1)y3p3+p−42+(p2+p+1)p−12yp3+p−2.

Let $G≅\langle x,y:x^{p^{2}}=y^{p}=1,y^{-1}xy=x^{p+1}\rangle .$ Then, for p≠2, $P\left(G\right)≅P\left(Z_{p}⋊Z_{p^{2}}\right)≅P\left(Z_{p}\times Z_{p^{2}}\right)$ and its reciprocal status Hosoya polynomial is given in Theorem 9. For p=2, $P\left(G\right)≅P\left(Z_{2}⋊Z_{2^{2}}\right)=K_{1}\vee \left(K_{3}\cup {\bar{K}}_{4}\right)$, then its Hosoya polynomial is

(8) $$4y^{11}+3y^{10}+3y^{9}.$$

Suppose $G≅\langle a,b,c:a^{p}=b^{p}=c^{p}=1,ac=cab,bc=cb,ab=ba\rangle .$ Then, for p≠2, $P\left(G\right)≅P\left(Z_{p}\times Z_{p}\times Z_{p}\right)≅P\left(Z_{p}⋊\left(Z_{p}\times Z_{p}\right)\right)$ and its reciprocal status Hosoya polynomial is devoted in Theorem 10. When p=2, then $P\left(G\right)≅P\left(Z_{2}⋊\left(Z_{2}\times Z_{2}\right)\right)≅P\left(Z_{2}⋊Z_{4}\right)$, and its reciprocal status is given by Equation (8).

The following result gives the power graph’s reciprocal status Hosoya polynomials of the cyclic group $Z_{pqr}.$

**Theorem** **11.** *Assume that $P(Z_{pqr})$ is the power graph of $Z_{pqr}$ of order pqr. Then*

Hrs(P(Zpqr),y)=1+pr−r−p2y2u1+(q−1)((p−1)(q−1)(r−1)+1)yu1+u2+(1+rq−r−q)(1+(p−1)(q−1)(r−1))yu1+u3+(r−1)((p−1)(q−1)(r−1)+1)yu1+u4+(1+pr−r−p)((q−1)(p−1)(r−1)+1)yu1+u5+(p−1)(1+(r−1)(q−1)(p−1))yu1+u6+(p−1)(1+(r−1)(q−1)(p−1))yu1+u7+q−12y2u2+(q−1)(qr−q−r+1)yu2+u3+(q−1)(pq−p−q+1)yu2+u7+1+rq−r−q2y2u3+(r−1)(1+rq−r−q)yu3+u4+r−12y2u4+(r−1)(rp−p−r+1)yu4+u5+1+pr−r−p2y2u5+(p−1)(1+pr−r−p)yu5+u6+p−12y2u6+(p−1)(1+pq−q−p)yu6+u7+1+pq−q−p2y2u7,

*where $u_{1}=pqr-1,\;u_{2}=\frac{2pqr-pr-1}{2},\;u_{3}=\frac{2pqr-pr-pq+p+q+r+1}{2},\;u_{4}=\frac{2pqr-pq-1}{2},\;u_{5}=\frac{1}{2}\left(2pqr-pq-qr+p+q+r-3\right),\;u_{6}=\frac{2pqr-qr-1}{2}$ and $u_{7}=\frac{2pqr-pr-qr+p+q+r-3}{2}.$*

**Proof.**  Using the node partitions of $P(Z_{pqr})$ as presented in Theorem 4, we obtain the following:

When, $u_{1}\in A_{1}$, implying $ec(u_{1})=1$, also, we use the reciprocal status concept, which results in the following:

$$rs(u_{1})=pqr-1.$$

When, $u_{2}\in A_{2}$, then $ec(u_{2})=2.$ Furthermore, we incorporate the concept of reciprocal status, which results in the following:

$$\begin{array}{cc} rs(u_{2})= & q-2+qrp-qp-rq-rp+q+p+r-1+1+rq-q-r+1+qp-q-p+1\\ \; & +\frac{1}{2}\left(p-1+pr-p-r+1+r-1\right)=\frac{2pqr-pr-1}{2}. \end{array}$$

When $u_{3}\in A_{3}$, then $ec(u_{3})=2;$ also, we use the reciprocal status concept, which results in the following:

$$\begin{array}{cc} rs(u_{3})= & qr-q-r+q-1+r-1+qrp-rq-rp-qp+r+q+p\\ \; & +\frac{1}{2}\left(pr-p-r+1+p-1+pq-p-q+1\right)\\ = & \frac{2pqr-pr-pq+p+q+r+1}{2}. \end{array}$$

When $u_{4}\in A_{4}$, it implies $ec(u_{4})=2.$ Furthermore, we incorporate the concept of reciprocal status, which results in the following:

$$rs\left(u_{4}\right)=\frac{2pqr-pq-1}{2}.$$

When $u_{5}\in A_{5}$, then $ec(u_{5})=2;$ furthermore, we use the idea of reciprocal status, resulting in the following:

$$rs\left(u_{5}\right)=\frac{2pqr-pq-qr+p+q+r-3}{2}.$$

When $u_{6}\in A_{6}$, then $ec(u_{6})=2;$ also, we use the reciprocal status concept, which results in the following:

$$rs\left(u_{6}\right)=\frac{2pqr-qr-1}{2}.$$

When $u_{7}\in A_{7}$, it implies $ec(u_{7})=2;$ also, we use the idea of reciprocal status, resulting in the following:

$$rs\left(u_{7}\right)=\frac{2pqr-pr-qr+p+q+r-3}{2}.$$

From the structure of $P(Z_{pqr})$, we see that there are 19 types of edges in $P(Z_{pqr})$ such as: $u_{1}∼u_{i},$ for i=1,2,3,4,5,6,7,$u_{2}∼u_{i},$ for i=2,3,4, $u_{3}∼u_{i},$ for i=3,4, $u_{4}∼u_{i},$ for i=4,5, $u_{5}∼u_{i},$ for i=5,6, $u_{i}∼u_{6}$, for i=6,7, and $u_{7}∼u_{7}$, where all $u_{i}$ values are as assigned above. Therefore,

(9) $$\begin{array}{cc} H_{rs}\left(P\left(Z_{pqr}\right)\right) & =\sum_{E_{u_{1}∼u_{1}}}y^{2u_{1}}+\sum_{E_{u_{1}∼u_{2}}}y^{u_{1}+u_{2}}+\sum_{E_{u_{1}∼u_{3}}}y^{u_{1}+u_{3}}+\sum_{E_{u_{1}∼u_{4}}}y^{u_{1}+u_{4}}+\sum_{E_{u_{1}∼u_{5}}}y^{u_{1}+u_{5}}\\ \; & +\sum_{E_{u_{1}∼u_{6}}}y^{u_{1}+u_{6}}+\sum_{E_{u_{1}∼u_{7}}}y^{u_{1}+u_{7}}+\sum_{E_{u_{2}∼u_{2}}}y^{2u_{2}}+\sum_{E_{u_{2}∼u_{3}}}y^{u_{2}+u_{3}}+\sum_{E_{u_{2}∼u_{7}}}y^{u_{2}+u_{7}}\\ \; & +\sum_{E_{u_{3}∼u_{3}}}y^{2u_{3}}+\sum_{E_{u_{3}∼u_{4}}}y^{u_{3}+u_{4}}+\sum_{E_{u_{4}∼u_{4}}}y^{2u_{4}}+\sum_{E_{u_{4}∼u_{5}}}y^{u_{4}+u_{5}}+\sum_{E_{u_{5}∼u_{5}}}y^{2u_{5}}\\ \; & +\sum_{E_{u_{5}∼u_{6}}}y^{u_{5}+u_{6}}+\sum_{E_{u_{6}∼u_{6}}}y^{2u_{6}}+\sum_{E_{u_{6}∼u_{7}}}y^{u_{6}+u_{7}}+\sum_{E_{u_{7}∼u_{7}}}y^{2u_{7}}. \end{array}$$

Moreover, the cardinality of $E_{u_{1}∼u_{1}}$ is pr−p−r+12. Similarly, we have:

|Eu1∼u2|=(q−1)(1+(r−1)(q−1)(p−1)),|Eu2∼u2|=q−12,|Eu3∼u3|=qr−q−r+12|Eu1∼u3|=(qr−q−r+1)(1+(r−1)(q−1)(p−1)),|Eu2∼u3|=(q−1)(1+rq−r−q)|Eu1∼u4|=(r−1)((p−1)(q−1)(r−1)+1),|Eu2∼u7|=(q−1)(pq−p−q+1)|Eu1∼u5|=(pr−p−r+1)((p−1)(q−1)(r−1)+1),|Eu3∼u4|=(r−1)(1+rq−r−q)|Eu1∼u6|=(p−1)(1+(p−1)(q−1)(r−1)),|Eu1∼u7|=(p−1)((p−1)(q−1)(r−1)+1),|Eu4∼u4|=r−12,|Eu4∼u5|=(r−1)(1+rp−r−p),|Eu5∼u5|=1+pr−r−p2,|Eu5∼u6|=(p−1)(pr−p−r+1),|Eu6∼u6|=p−12,|Eu6∼u7|=(p−1)(1+qp−q−p),|Eu7∼u7|=1+qp−q−p2.

Putting all these values in Equation (9), we obtain the required reciprocal status Hosoya polynomial. □

**Theorem** **12.** *Suppose P(G) is the power graph of $G=Z_{r}\times F_{p,q}$ of order pqr. Then*

Hrs(P(G),y)=(p−1)yu1+u2+(pr−p−r+1)yu1+u3+(r−1)yu1+u4+p(qr−q−r+1)yu1+u5+p(q−1)yu1+u6+q−12y2u2+(1+pr−r−p)(p−1)yu2+u3+r−12y2u4+1+pr−r−p2y2u3+(r−1)(1+pr−r−p)yu3+u4+1+qr−r−q2y2u5+p(r−1)(1+qr−r−q)yu4+u5+p(q−1)(qr−q−r+1)yu5+u6+q−12y2u6,

*where $u_{1}=pqr-1,\;u_{2}=\frac{1}{2}\left(pqr+pr-r-1\right),\;u_{3}=\frac{1}{2}\left(pqr+pr-2\right),\;u_{4}=\frac{1}{2}\left(2pqr-pq-1\right),\;u_{5}=\frac{1}{2}\left(pqr+qr-2\right),$ and $u_{6}=\frac{1}{2}\left(pqr+qr-r-1\right)$.*

**Proof.**  We obtain the following from Figure 1 and the partitions defined in Theorem 5:

When the node $u_{1}=e$, then $ec(u_{1})=1$; furthermore, we incorporate the concept of reciprocal status, which results in the following:

$$rs(u_{1})=pqr-1.$$

When $u_{2}\in A_{2}$, it implies $ec(u_{2})=2;$ also, we incorporate the concept of reciprocal status, which results in the following:

$$\begin{array}{cc} rs(u_{2})= & \;\frac{1}{2}\left(+p(q-1)+(r-1)+p(1+qr-r-q)\right)+p-2+1+pr-p-r+1\\ = & \;\frac{1}{2}\left(pqr+pr-r-1\right). \end{array}$$

When $u_{3}\in A_{3}$, then $ec(u_{3})=2;$ also, we use the reciprocal status concept, which results in the following:

$$\begin{array}{c} rs\left(u_{3}\right)=\frac{1}{2}\left(2pqr-pq-1\right). \end{array}$$

When $u_{4}\in A_{4}$, it implies $ec(u_{4})=2;$ further, we incorporate the concept of reciprocal status, which results in the following:

$$rs\left(u_{4}\right)=\frac{1}{2}\left(pqr+qr-2\right).$$

When $u_{5}\in A_{5}$, then $ec(u_{5})=2;$ additionally, we incorporate the concept of reciprocal status, which results in the following:

$$rs\left(u_{5}\right)=\frac{1}{2}\left(pqr+qr-r-1\right).$$

From the structure of P(G), we see that there are 14 kinds of edges in P(G), such as: $u_{1}∼u_{i},$ for i=2,3,4,5,6,$u_{2}∼u_{i},$ for i=2,3, $u_{3}∼u_{i},$ for i=3,4, $u_{4}∼u_{i},$ for i=4,5, $u_{5}∼u_{i},$ for i=5,6, $u_{6}∼u_{6}$, where $u_{i}$ values are assigned as above. Therefore:

(10) $$\begin{array}{cc} H_{rs}\left(P\left(G\right),y\right)= & \sum_{E_{u_{1}∼u_{2}}}y^{u_{1}+u_{2}}+\sum_{E_{u_{1}∼u_{3}}}y^{u_{1}+u_{3}}+\sum_{E_{u_{1}∼u_{4}}}y^{u_{1}+u_{4}}+\sum_{E_{u_{1}∼u_{5}}}y^{u_{1}+u_{5}}+\sum_{E_{u_{1}∼u_{6}}}y^{u_{1}+u_{6}}\\ \; & +\sum_{E_{u_{2}∼u_{2}}}y^{2u_{2}}+\sum_{E_{u_{2}∼u_{3}}}y^{u_{2}+u_{3}}+\sum_{E_{u_{3}∼u_{3}}}y^{2u_{3}}+\sum_{E_{u_{3}∼u_{4}}}y^{u_{3}+u_{4}}+\sum_{E_{u_{4}∼u_{4}}}y^{2u_{4}}\\ \; & +\sum_{E_{u_{4}∼u_{5}}}y^{u_{4}+u_{5}}+\sum_{E_{u_{5}∼u_{5}}}y^{2u_{5}}+\sum_{E_{u_{5}∼u_{6}}}y^{u_{5}+u_{6}}+\sum_{E_{u_{6}∼u_{6}}}y^{2u_{6}}. \end{array}$$

Now, the cardinality of $E_{u∼v}$ values are

|Eu1∼u2|=p−1,|Eu1∼u3|=pr−p−r+1,|Eu1∼u4|=r−1,|Eu1∼u5|=p(qr−q−r+1),|Eu1∼u6|=p(q−1),|Eu2∼u2|=q−12,|Eu2∼u3|=(p−1)(1+pr−r−p),|Eu3∼u3|=1+pr−r−p2,|Eu3∼u4|=(r−1)(1+pr−r−p),|Eu4∼u4|=r−12,|Eu4∼u5|=p(1+qr−r−q)(r−1),|Eu5∼u5|=1+qr−q−r2|Eu5∼u6|=p(1+qr−r−q)(q−1),|Eu6∼u6|=q−12.

Putting all these values in Equation (10), we obtain the required result. □

Following the procedure as used in the above theorems, we obtain the following results.

**Theorem** **13.** *Let $P(F_{p,qr}),$ where r=3 or q=3 is a power graph of $F_{p,qr}$ of order pqr. Then*

Hrs(P(Fp,qr),y)=(p−1)yu1+u2+(pr−p−r+1)yu1+u3+(r−1)yu1+u4+p(qr−r)yu1+u5+p−12y2u2+(p−1)(1+pr−r−p)yu2+u3+1+pr−r−p2y2u3+(r−1)(1+pr−r−p)yu3+u4+r−12y2u4+p(qr−r)(r−1)yu4+u5+pqr−r2y2u5.

*where $u_{1}=pqr-1,\;u_{2}=\frac{1}{2}\left(pqr+pr-r-1\right),\;u_{3}=\frac{1}{2}\left(pqr+pr-2\right),\;u_{4}=\frac{1}{2}\left(2pqr-2r+p-1\right),$ and $u_{5}=\frac{1}{2}\left(pqr+qr-2\right)$.*

**Theorem** **14.** *Suppose $P(F_{p,qr})$ is the power graph of $F_{p,qr}$ of order pqr. Then*

Hrs(P(Fp,qr),y)=(p−1)yu1+u2+p(qr−1)yu1+u3+p−12y2u2+pqr−12y2u3,

*where $u_{1}=pqr-1,\;u_{2}=\frac{1}{2}\left(pqr+p-2\right),$ and $u_{3}=\frac{1}{2}\left(pqr+qr-2\right).$*

**Theorem** **15.** *Let $P(G_{i+5})$ be the power graph of a group $G_{i+5}$ of order pqr. Then*

Hrs(Gi+5,y)=(p−1)yu1+u2+(pr−p−r+1)yu1+u3+(q−1)yu1+u4+pq(r−1)yu1+u5+p−12y2u2+(p−1)(1+pr−r−p)yu2+u3+1+pr−r−p2y2u3+(q−1)(1+pq−q−p)yu3+u4+q−12y2u4+pqr−12y2u5.

*where $u_{1}=pqr-1,\;u_{2}=\frac{1}{2}\left(pqr+pq-q-1\right),\;u_{3}=\frac{1}{2}\left(pqr+pq-2\right),\;u_{4}=\frac{1}{2}\left(pqr+pq-p-1\right),$ and $u_{5}=\frac{1}{2}\left(pqr+r-2\right)$.*

## 5. Conclusions

The main objective of this article was to examine the structural characteristics of the power graphs of finite abelian and non-abelian groups. In general, finding the (reciprocal status) Hosoya polynomials of graphs is very difficult. The researchers try to study the same for different classes of graphs. The (reciprocal status) Hosoya polynomials of graphs defined on algebraic structures have attracted the attention of researchers. In this paper, we made a little effort and discussed the Hosoya polynomials as well as the reciprocal status Hosoya polynomials of the power graphs associated with finite groups of order pq and pqr.

However, the (reciprocal status) Hosoya polynomials for general power graphs are open and remain a challenge. In chemistry, an algebraic structure is critical for forming chemical structures and investigating the different chemical characteristics of chemical compounds included inside these structures.

## Data Availability

The data used to support the findings of this study are available within the article.

## Sample Availability

Samples of the compounds are not available from the authors.
